# Klotho and S100A8/A9 as Discriminative Markers between Pre-Renal and Intrinsic Acute Kidney Injury

**DOI:** 10.1371/journal.pone.0147255

**Published:** 2016-01-22

**Authors:** Ae Jin Kim, Han Ro, Hyunsook Kim, Jae Hyun Chang, Hyun Hee Lee, Wookyung Chung, Ji Yong Jung

**Affiliations:** 1 Division of Nephrology, Department of Internal Medicine, Gachon University Gil Medical Center, Gachon University School of Medicine, Incheon, Korea; 2 Gachon Medical Research Institute, Gachon University Gil Medical Center, Incheon, Korea; Hospital Universitario de La Princesa, SPAIN

## Abstract

Early detection and accurate differentiation of the cause of AKI may improve the prognosis of the patient. However, to date, there are few reliable biomarkers that can discriminate between pre-renal and intrinsic AKI. In this study, we determined whether AKI is associated with altered serum and urinary levels of Klotho, S100A8/A9 (an endogenous ligand of toll-like receptor 4), and neutrophil gelatinase-associated lipocalin (NGAL), which may allow differentiation between pre-renal and intrinsic AKI. A volume-depleted pre-renal AKI model was induced in male Sprague Dawley rats fed a low-salt diet (0.03%) without water 96 h before two intraperitoneal (IP) injections of furosemide (20 mg/kg) at a 24 h interval. In contrast, in the cisplatin-induced intrinsic AKI model, animals were given a single IP injection of cisplatin (5 mg/kg). All of the animals were euthanized 72 h after the first IP injection. Serum and urinary levels of Klotho, S100A8/A9, and NGAL were measured using an enzyme-linked immunosorbent assay. We also performed a proof-of-concept cross-sectional study to measure serum and urinary biomarkers in 61 hospitalized patients with established AKI. Compared to the intrinsic AKI group, the pre-renal AKI group showed a marked depression in urinary Klotho levels (13.21±17.32 vs. 72.97±17.96 pg/mL; P = 0.002). In addition, the intrinsic AKI group showed marked elevation of S100A8/A9 levels compared to the pre-renal AKI group (2629.97±598.05 ng/mL vs. 685.09±111.65 ng/mL; P = 0.002 in serum; 3361.11±250.86 ng/mL vs. 741.72±101.96 ng/mL; P = 0.003 in urine). There was no difference in serum and urinary NGAL levels between the pre-renal and intrinsic AKI groups. The proof-of-concept study with the hospitalized AKI patients also demonstrated decreased urinary Klotho in pre-renal AKI patients and increased urinary S100A8/A9 concentrations in intrinsic AKI patients. The attenuation of urinary Klotho and increase in urinary S100A8/A9 may allow differentiation between pre-renal and intrinsic AKI.

## Introduction

Acute kidney injury (AKI) is a serious problem associated with high morbidity and mortality [1]. Despite remarkable progress in medical care, the incidence of AKI in hospitalized patients remains high [2]. The prognosis of AKI depends crucially on the early and correct identification of the underlying cause of the disease and the immediate onset of therapy [3]. To date, it has been considered reliable to use serum creatinine for the diagnosis of AKI, but it is a somewhat inadequate gold standard for many reasons. Serum creatinine has poor specificity, because it is affected by age, gender, muscle mass, dietary intake, and medications, all of which may lead to changes in serum creatinine without actual kidney injury [4]. In addition, serum creatinine may not change despite real tubular injury, because other nephrons may have an adequate compensatory renal reserve [5]. The use of serum creatinine may also cause delays in diagnosis and treatment because serum creatinine tends to increase slowly after injury [6]. Thus, there has been recent interest in identifying novel AKI biomarkers for early diagnosis and risk stratification.

The various causes of AKI are commonly classified according to their origin as pre-renal, intrinsic, and post-renal. Whereas post-renal AKI is readily diagnosed by imaging studies, to date, there has been no reliable tool for differentiating between pre-renal and intrinsic AKI. When renal dysfunction is improved within 24–72 h solely by fluid resuscitation, it is usually considered that the patient has had pre-renal AKI. However, waiting to identify volume responsiveness is unacceptable in cases of crescentic glomerulonephritis, which require immediate diagnosis and treatment or acute tubular necrosis. Moreover, fluid resuscitation can endanger non-volume depleted patients and may lead to poor AKI outcomes, including mortality [7]. Fractional excretion of sodium (FENa) is another index for differentiating between pre-renal and intrinsic AKI. Although FENa is widely used, its sensitivity and specificity are significantly decreased in patients with underlying chronic kidney disease, heart failure, liver cirrhosis, and sepsis, and with the use of diuretics.

A reliable non-invasive marker for discriminating between pre-renal and intrinsic AKI is desirable for its early differential diagnosis and appropriate treatment, which would improve outcomes in AKI patients. However, there have been few studies on discriminative markers for AKI. Previous research has shown that rat *klotho* mRNA expression is markedly decreased by acute inflammatory stress, but not by hypovolemic stress [8]. Thus, we presume that *klotho* might differentiate between functional loss and structural damage in the kidney. S100A8/A9, an activator of the innate immune system, is increased in various inflammatory disorders [9]. Recent studies have shown that inflammatory responses concerned with the innate and adaptive immune systems contribute considerably to parenchymal damage in AKI [10]. Thus, we presume that S100A8/A9 may be elevated in intrinsic AKI due to the increased inflammatory response, whereas it may not be elevated in pre-renal AKI due to the absence of epithelial damage and inflammation. This might allow discrimination between pre-renal and intrinsic AKI. Neutrophil gelatinase-associated lipocalin (NGAL) is one of the most widely studied novel biomarkers in the pathophysiology of AKI. A recent study reported that urinary NGAL concentrations were higher in patients with intrinsic AKI than those with pre-renal AKI [11,12]. Thus, we attempted to identify the discriminative function of NGAL between pre-renal and intrinsic AKI.

This study was conducted to evaluate new AKI biomarkers, including serum and urinary levels of Klotho, S100A8/A9, and NGAL, for differentiating between pre-renal and intrinsic AKI. We also conducted a proof-of-concept study to determine if biomarkers effective for animal models can also differentiate between pre-renal and intrinsic AKI in hospitalized AKI patients.

## Materials and Methods

### Experimental Animals

Eighteen male Sprague Dawley rats were purchased from Orient Bio (Seongnam City, Gyeonggi Province, Korea). Animals were checked daily for any signs of sickness during the experimental period. All of the animal experiments were performed under the approval of the Institutional Animal Care and Use Committee of the Clinical Research Institute at Gachon University Gil Medical Center, and in accordance with the Guidelines for the Care and Use of Laboratory Animals of the Gachon University Lee Gil Ya Cancer and Diabetes Institute.

### Induction of AKI

**Fig 1:**
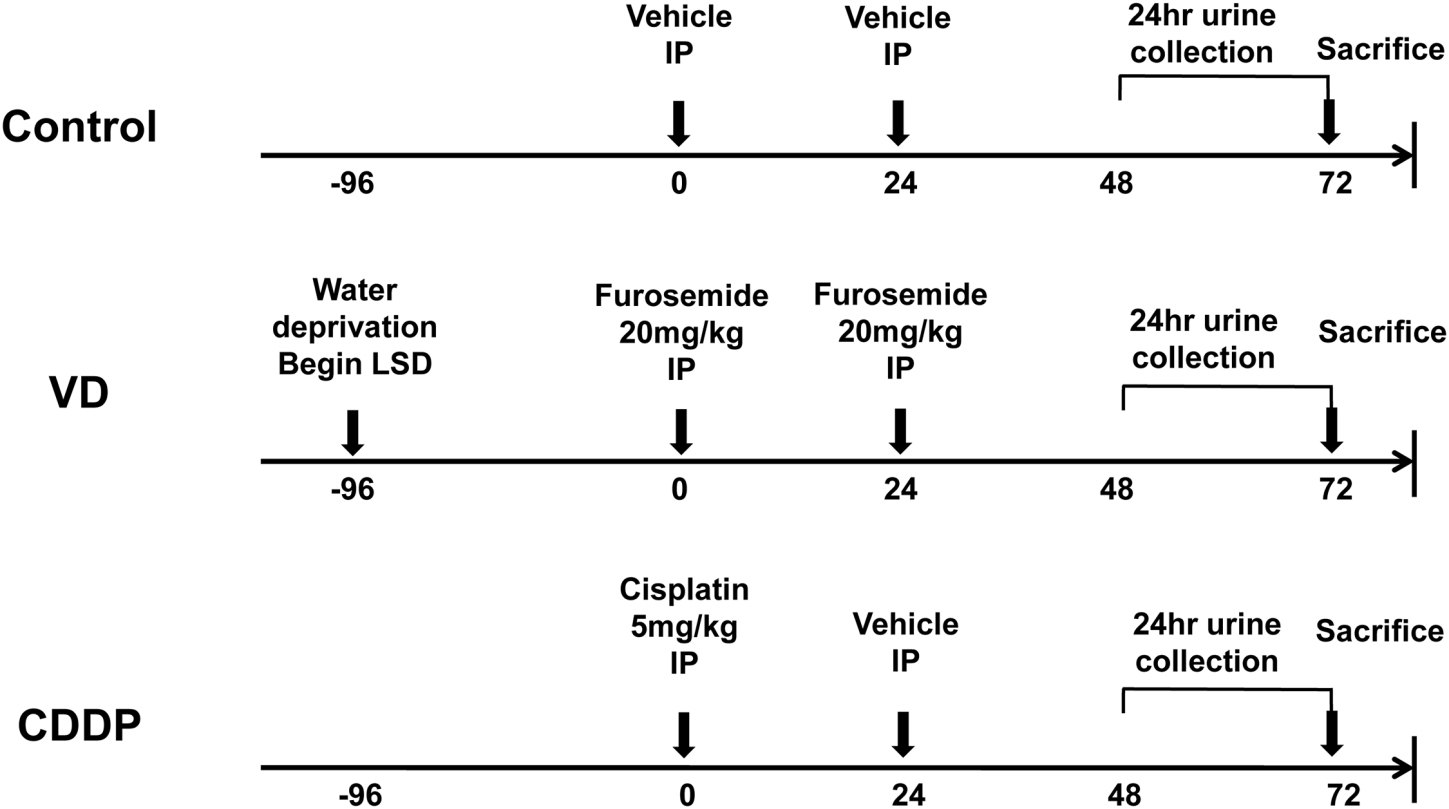
Experimental scheme. A volume-depleted pre-renal AKI model (VD) was induced in male Sprague Dawley rats fed a low-salt diet (0.03%) without water 96 h before two intraperitoneal (IP) injections of furosemide (20 mg/kg) at a 24 h interval. In contrast, in the cisplatin-induced intrinsic AKI model (CDDP), animals were given a single IP injection of cisplatin (5 mg/kg). All of the animals were euthanized 72 h after the first IP injection.

Rats were distributed evenly into the following three groups (Fig 1).

1. Volume depletion (VD) (*n* = 6; 306–347 g; 8 weeks old): Rats were maintained on a low salt diet (0.03% NaCl; Harlan Teklad, Cambridgeshire, UK) and water deprivation from 96 h before furosemide injection. Intraperitoneal (IP) injections of furosemide (20 mg/kg) were administered at 0 and 24 h.
2. Cisplatin-induced AKI (cis-diamminedichloroplatinum; CDDP) (*n* = 6; 306–316 g; 8 weeks old): Rats had free access to water and food (PicoLab Rodent Diet 20 5053; LabDiet, St. Louis, MO, USA). A single IP injection of CDDP (5 mg/kg) (Cisplan Inj 25 mg/20 mL; Dong-A ST, Seoul, Korea) was given at 0 h, and a single IP of vehicle (normal saline) was given at 24 h.
3. Control (*n* = 6; 302–314g; 8 weeks old): Rats had free access to water and food (PicoLab Rodent Diet 20 5053; LabDiet, St. Louis, MO, USA). Two IP injections of vehicle (normal saline) were administered at 0 h and 24 h.

Previously described experimental models of VD and CDDP-induced AKI models were modified in this study [13,14]. All of the animals were placed in metabolic cages, and 24 h urine samples were collected 48–72 h after the first injection with furosemide, CDDP, or normal saline. Rats were fully anesthetized by isoflurane inhalation before blood sampling and phlebotomy. Blood samples were collected from the abdominal aorta at 72 h after the first injection of furosemide, CDDP, or normal saline. After blood sampling, rats were sacrificed by phlebotomy and both kidneys were removed. Kidneys were promptly fixed in 10% buffered formalin solution.

### Measurements of serum and urinary biochemical parameters

The blood samples were centrifuged (2500 rpm, 15 min). The serum was immediately frozen at -80°C. Blood urea nitrogen (BUN) and creatinine concentrations were measured by the kinetic Jaffé-method (Modular Analytics; Roche Diagnostics, Mannheim, Germany). Electrolytes were measured by indirect ion-selective electrode measurements (Modular Analytics; Roche Diagnostics, Mannheim, Germany). The total urine volume was documented, and the samples were centrifuged (900×*g*, 10 min). Then, 5 mL urine was promptly frozen at -80°C. Samples were thawed and centrifuged (10,000×*g*, 10 min, 4°C) at the time of analysis. Urine urea nitrogen was determined with a Cobas Integra 800 (Roche Diagnostics, Mannheim, Germany), and urine creatinine and sodium levels were determined using Modular Analytics (Roche Diagnostics, Mannheim, Germany). FENa ([urine sodium × plasma creatinine] / [plasma sodium × urine creatinine] × 100) and FEUrea ([urine urea × plasma creatinine] / [serum urea × urine creatinine] × 100) were calculated from the measurements.

### Examination of biomarkers

#### Enzyme-linked immunosorbent assay

Klotho (Rat Klotho ELISA Kit; Cusabio Biotech, Wuhan, China), S100A8/A9 (Calprotectin ELISA Kit; MyBioSource, San Diego, CA, USA), and NGAL (Lipocalin-2 Rat ELISA Kit; abcam, Cambridge, UK) levels were assessed by enzyme-linked immunosorbent assay (ELISA) according to the manufacturer’s instructions. Because variability in urine concentrations could impair the use of a urinary biomarker as a discriminative marker, we corrected biomarker concentrations with reference to the urinary creatinine concentrations.

#### Semiquantitative immunoblotting

The right kidneys were dissected into small pieces and placed in chilled radioimmunoprecipitation assay (RIPA) buffer (iNtRON biotechnology, Seongnam-si, Korea) containing with protease inhibitor cocktail (Sigma-aldrich, St. Louis, MO, USA). The total protein concentration was measured by the bicinchoninic acid protein assay method (Pierce BCA protein assay kit; Thermo scientific, Rockford, IL, USA). The protein lysates were denatured by boiling in sodium dodecyl sulfate (SDS) sample buffer at 94°C for 5 min and were then centrifuged at 15,000 × g for 15 min at 4°C. Next, the supernatant was separated on SDS-polyacrylamide gels. Separated protein lysates (klotho 15ug, NGAL 7ug, calprotectin 60ug) were electrophoresed and transferred to polyvinylidene difluoride (PVDF) membranes (Merck Millipore, Darmstadt, Germany). The membranes were blocked with 5% DifcoTM Skim milk (BD, Sparks, MD, USA) and incubated with primary antibodies overnight at 4°C. Primary antibodies used in this study were anti-Klotho (1:1000, abcam, Cambridge, UK), anti-Lipocalin 2 (1:1000, abcam, Cambridge, UK), anti-Calgranulin A (S100A8; 1:100, Santa Cruz Biotechnology, Dallas, TX, USA), anti-Calgranulin B (S100A9; 1:100, Santa Cruz Biotechnology, Dallas, TX, USA), and anti-β-actin (1:2000, Santa Cruz Biotechnology, Dallas, TX, USA). The membranes were washed three times with TBST (Tris-buffered saline containing 0.05% Tween 20) and incubated with horseradish peroxidase (HRP) conjugated secondary antibodies (1:5000) for 1 h at room temperature. Secondary antibodies used in this study were anti-mouse IgG-HRP, anti-rabbit IgG-HRP, and anti-goat IgG-HRP (Santa Cruz Biotechnology, Dallas, TX, USA). The immunoreactive bands were visualized with an enhanced chemiluminescence solution (EzWestLumi plus; ATTO, Osaka, Japan) using the ImageQuant LAS 4000 camera system (GE Healthcare Life Sciences, Little Chalfont, UK). The intensity of each band was determined using ImageJ software (version 1.49; National Institutes of Health, Bethesda, MD, USA).

#### Histological analysis

To assess the light microscopic appearance, 4μm thick paraffin sections were subjected to hematoxylin and eosin (H&E) staining. H&E-stained kidney sections were photographed by one of the investigators blinded to the experimental protocol using an Olympus BX51 microscope. Previously described semi-quantitative pathological scoring system was used with minor modification [15,16]. The severity of renal damage was scored with a grading system (0, 1, 2, 3): 0 = no visible lesions, normal or near normal kidney morphology; 1 = mild dilation in some tubules, cell swelling, luminal debris (cast), and nuclear condensation, partial loss of brush border membranes in < 1/3 tubules in high field; 2 = obvious dilation of many tubules, loss of brush border membranes, nuclear loss, and cast in < 2/3 tubules in high field; 3 = severe dilation of most tubules, total loss of brush border membranes, and nuclear loss in > 2/3 tubules in high field. Fifty fields (400×) were counted for cortex and outer stripe of outer medulla in each kidney section. The total score for each kidney was calculated by addition of all scores from 100 fields with a maximum score of 300.

#### Immunohistochemistry

For the immunohistochemistry study, paraffin wax-embedded kidney was cut into 4 μm sections. Xylene and an ethanol series were used for deparaffinization and hydration, respectively. After blocking with 5% bovine serum albumin (BSA) solution for 1h, sections were then incubated at 4°C overnight with the following primary antibodies: anti-Klotho antibody (1:200, abcam, Cambridge, UK), anti-MRP8+MRP14 antibody (S100A8+S100A9; 1:200, abcam, Cambridge, UK), and anti- Lipocalin2 antibody (1:200, abcam, Cambridge, UK). Following washing with TBST, streptavidin-biotin/HRP was applied (LSAB+System-HRP kit, Dako, Carpinteria, CA, USA). Sections were counterstained with Mayer’s hematoxylin.

#### Confocal microscopy

Confocal microscopy was performed with a LSM 700 laser scanning confocal microscope (Carl Zeiss; Jena, Germany). After blocking with 5% BSA solution for 1h, sections were incubated at 4°C overnight with the following primary antibodies: anti-MRP8+MRP14 (1:200, abcam, Cambridge, UK), anti-CD15 (1:200, Bioss, Woburn, MA, USA), and anti CD-68 (1:200, abcam, Cambridge, UK) antibodies. A second layer of Alexa Fluor 488-conjugated anti-mouse IgG antibody (1:1000, abcam, Cambridge, UK) and TRITC-conjugated anti-rabbit IgG antibody (1:1000, abcam, Cambridge, UK) were used as secondary antibodies. Sections were counterstained with 4’-6-diamidino-2-phenylindole (DAPI; Vector, Burlingame, CA, USA).

### Human Study

#### Study Design and Participants

This study was performed as a cross-sectional study. Adult (aged over 18 years old) patients with AKI who had been admitted to the general ward or intensive care units in Gachon University Gil Medical Center between January and December 2014 were eligible for enrollment. AKI was defined as a rapid (within 48 h) decline in renal function, designated as an absolute increase in serum creatinine of at least 0.3 mg/dL, a percentage increase in serum creatinine of at least 50%, or documented oliguria of less than 0.5 mL/kg/h for more than 6 h [17]. Patients were excluded for post-renal obstruction as a cause of AKI, previous history of RRT, or renal transplantation. Patients were considered to have pre-renal AKI when the rise in serum creatinine concentration had been caused by factors that reduce renal perfusion, such as dehydration, bleeding, hepatorenal syndrome, and cardiorenal syndrome, and when creatinine recovered rapidly to baseline after volume repletion within 3 days. Recovery from AKI was defined by the improvement of creatinine to baseline levels in patients who have followed this hospital with known baseline creatinine. In case of in-hospital AKI, reverse decrease of creatinine from AKIN classification (absolute decrease in serum creatinine of at least 0.3mg/dL or a percent decrease in serum creatinine of at least 50%) or to the level of before AKI occurrence was considered to recover from AKI. In addition, patients with pre-renal AKI had no previous exposure history to insults that result in intrinsic kidney injury. Patients were considered to have intrinsic AKI when the increased serum creatinine concentration did not respond to fluid replacement within 3 days and/or was associated with nephrotoxic events, such as hypotension, sepsis, interstitial nephritis, contrast-induced AKI, and glomerulonephritis.

The study was approved by the Institutional Review Board at the Gachon University Gil Medical Center (GAIRB2014–202). Written informed consent was obtained from all of the patients. The biospecimens and data used were provided by Gachon University Gil Medical Center Bio Bank (No: GBB2014–04).

#### Parameters

We obtained the following demographic data: age, gender, comorbid conditions (diabetes, hypertension, cardiovascular disease), and medication history (ACE inhibitors/ARBs, statins, β-blockers, calcium channel blockers, diuretics, aspirin). AKI severity by the Risk, Injury, Failure, Loss, and End-stage kidney (RIFLE) and Acute Kidney Injury Network (AKIN) criteria were estimated on the day of the serum and urine sample. Serum and urinary Klotho (Human soluble α-Klotho Assay Kit—IBL; IBL, Gunma, Japan), S100A8/A9 (PhiCal Calprotectin ELISA Kit; Immundiagnostik AG, Bensheim, Germany), and NGAL (Lipocalin-2 Human ELISA Kit; abcam, Cambridge, UK) levels were assessed by ELISA according to the manufacturer’s instructions. Biomarker concentrations were corrected with reference to the urinary creatinine concentrations.

#### Statistical analyses

Continuous data are reported as means ± standard deviation (SD) unless otherwise specified. Categorical data are reported as absolute values and percentage. Non-parametric tests of significance were performed. The Kruskal-Wallis test with Dunn’s multiple comparisons was used when comparing three groups. The Mann-Whitney U-test for continuous variable and Fisher’s exact test for categorical variables were used when comparing two groups. Statistical analyses were performed using the GraphPad Prism software (ver. 6.0; GraphPad, San Diego, CA, USA). The significance level was set at P < 0.05.

## Results

### Animal study

#### Physiologic parameters

None of the animals died during the experimental period. Body weight significantly declined in the VD group (from 318.33±15.20 to 215.83±13.00 g, 32.4%, P = 0.027), and increased in the control group (from 307.17±4.54 to 330.67±14.25 g, 7.1%, P = 0.028). There was no marked change in body weight in the CDDP group (from 310.67±4.27 to 313.00±11.58 g, 0.64%, P = 0.753; Table 1). Urine output during the 24 h before sacrifice was significantly decreased in the VD group (Control, 10605.0±2299.56 μL; VD, 3608.33±865.11 μL; CDDP, 18453.33±7961.99 μL; P = 0.001; Table 1).

**Table 1:** Parameters of acute kidney injury animal models.

	Control	Volume depletion induced AKI	Cisplatin induced AKI	*P*
	(*n* = 6)	(*n* = 6)	(*n* = 6)	
Body weight, initial (g)	307.17±4.54	318.33±15.20	310.67±4.27	0.134
Body weight, final (g)	330.67±14.25	215.83±13.00*	313.00±11.58	**0.002**
Urine output, final (μL)	10605.0±2299.56	3608.33±865.11	18453.33±7961.99†	**0.001**
Creatinine (mg/dL)	0.24±0.05	0.62±0.26*	0.65±0.23*	**0.004**
Blood urea nitrogen (mg/dL)	19.17±2.06	98.38±68.51*	34.73±26.40	**0.003**
Serum Na (mmol/L)	146.00±2.61	143.50±15.07	144.83±2.48	0.204
FeNa (%)	0.28±0.06	0.04±0.02	0.96±0.61†	**0.001**
FeUrea (%)	25.39±5.09	27.08±9.89	39.54±14.16	0.087
Urine creatinine (mg/dL)	106.38±23.93	159.85±52.65	60.52±24.07†	**0.002**
Urine urea nitrogen (mg/dL)	2174.33±409.57	5844.83±2240.85	1093.17±514.10†	**0.001**
Urine Na (mmol/L)	183.17±46.18	13.83±3.55*	124.00±54.79	**0.001**

Abbreviations: AKI, acute kidney injury, FeNa, fractional excretion of sodium, FeUrea, fractional excretion of urea.

*, p < 0.05, vs. control group.

^†^, p < 0.05, vs. VD group.

Statistical analyses were performed with the Kruskal-Wallis test followed by Dunn’s multiple-comparisons test.

#### Induction of AKI

Results of the serum and urinary biochemical parameters are shown in Table 1. Serum creatinine increased similarly in the VD and CDDP groups compared to the control group (Control, 0.24±0.05 mg/dL; VD, 0.62±0.26 mg/dL; CDDP, 0.65±0.23 mg/dL, P = 0.004; Table 1). Blood urea nitrogen level was elevated in the VD group compared to the CDDP and control groups (Control, 19.17±2.06 mg/dL; VD, 98.38±68.51 mg/dL; CDDP, 34.73±26.40 mg/dL; P = 0.003; Table 1). FENa, considered to increase in intrinsic AKI, was increased in the CDDP group versus the VD and control groups, as expected (Control, 0.28±0.06%; VD, 0.04±0.02%; CDDP, 0.96±0.61%; P = 0.001; Table 1). FEUrea also showed a tendency to increase in the CDDP group versus the VD and control groups, although the difference was not statistically significant (Control, 25.39±5.09%; VD, 27.08±9.89%; CDDP, 39.54±14.16%; P = 0.087; Table 1).

#### Serum and urinary levels of Klotho, S100A8/A9, and NGAL for differentiating between pre-renal and intrinsic AKI models

**Fig 2:**
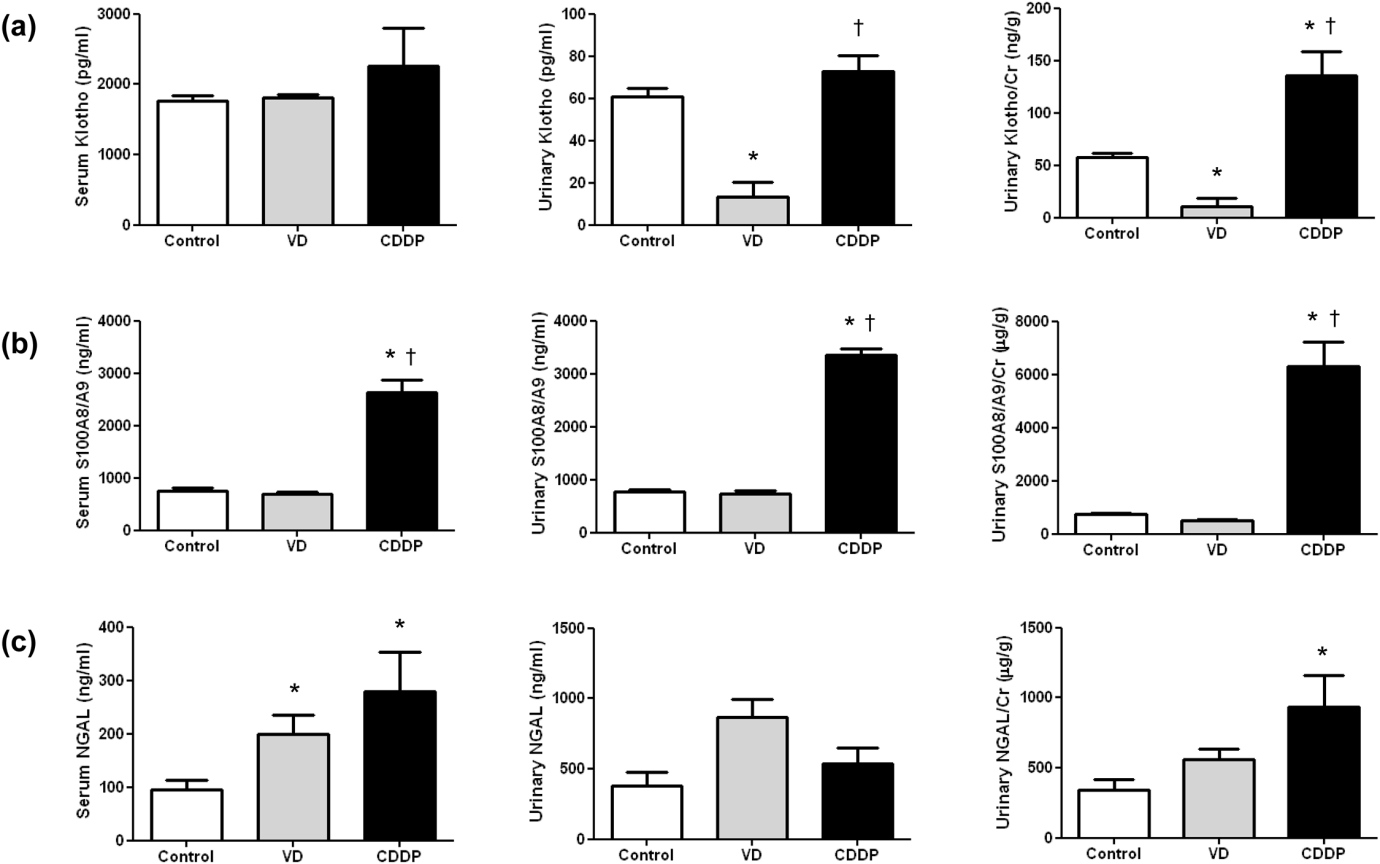
Serum and urinary Klotho, S100A8/A9, and Neutrophil gelatinase-associated lipocalin (NGAL) concentrations in an acute kidney injury model. (a) Urinary Klotho and urinary Klotho/creatinine (Cr) decreased significantly in the volume depletion (VD) group. (b) Serum and urinary S100A8/A9 and urinary S100A8/A9/Cr increased significantly in the cisplatin (CDDP) group. (c) Serum and urinary NGAL showed no significant difference between the VD and CDDP groups. Data are given as means±SD. (*n* = 6 for each group). *, p < 0.05, vs. the control group; †, p < 0.05, vs. the VD group.

Serum Klotho levels did not significantly differ among the three groups (Control, 1770.09±168.72 pg/mL; VD, 1814.51±109.33 pg/mL; CDDP, 2264.02±1298.88 pg/mL; P = 0.692). Urinary Klotho and Klotho/creatinine levels were significantly decreased in the VD group (urine Klotho, 13.22±17.31 pg/mL; urine Klotho/creatinine, 11.15±17.83 ng/g) compared to the CDDP group (urine Klotho, 72.97±17.96 pg/mL; urine Klotho/creatinine, 135.43±57.61 ng/g) (urine Klotho, P = 0.004; urine Klotho/creatinine, P = 0.001; Fig 2(a)).

Serum and urine levels of S100A8/A9 and S100A8/A9/creatinine were significantly higher in the CDDP group (serum S100A8/A9, 2629.97±598.05 ng/mL, urine S100A8/A9, 3361.11±250.86 ng/mL, urine S100A8/A9/creatinine, 6292.21±2303.06 μg/g) than the VD (serum S100A8/A9, 685.09±111.65 ng/mL, urine S100A8/A9, 741.72±101.96 ng/mL, urine S100A8/A9/creatinine, 490.83±109.05 μg/g) and control groups (serum S100A8/A9, 754.79±131.69 ng/mL, urine S100A8/A9, 765.96±94.85 ng/mL, urine S100A8/A9/creatinine, 743.46±153.58 μg/g), (serum S100A8/A9, P = 0.002; urine S100A8/A9, P = 0.003; urine S100A8/A9/creatinine, P = 0.001; Fig 2(b)).

Serum NGAL levels in the CDDP and VD groups were higher than those in the control group (Control, 95.73±45.48 ng/mL; VD, 198.94±87.54 ng/mL; CDDP, 279.70±178.95 ng/mL; P = 0.006). Urinary NGAL did not significantly differ among three groups (Control, 376.28±256.70 ng/mL; VD, 868.15±308.08 ng/mL; CDDP, 537.63±276.93 ng/mL; P = 0.065). Urine NGAL/creatinine levels were higher in the CDDP group than in the control group, but not than the VD group (Control, 342.66±183.72 μg/g; VD, 559.75±187.12 μg/g; CDDP, 931.70±558.51 μg/g; P = 0.009; Fig 2(c)).

#### Histology and semiquantitative pathologic score of AKI models

**Fig 3:**
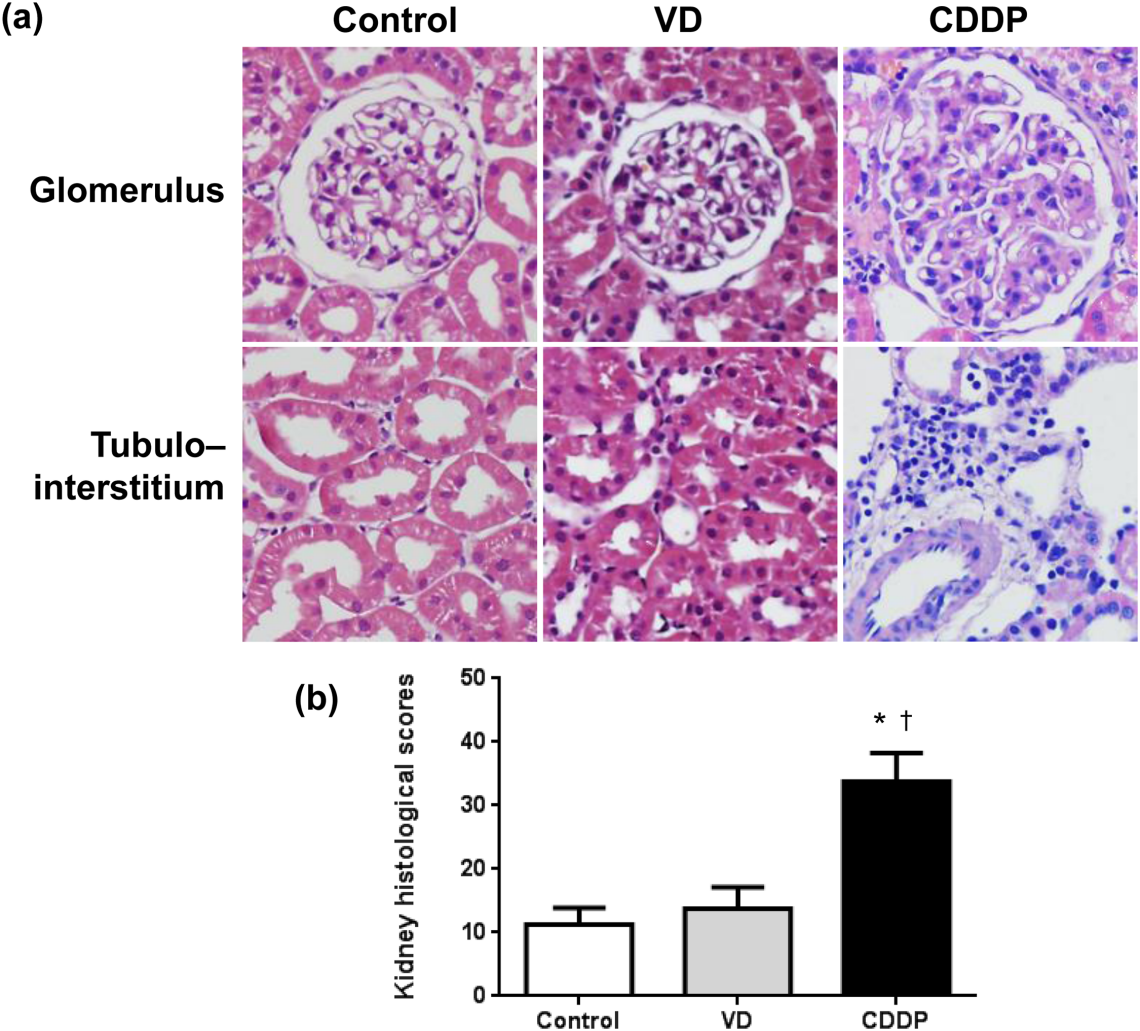
Representative hematoxylin and eosin (H&E) staining and renal pathological score of the acute kidney injury model. (a) H&E staining showed near normal or mild brush border loss in the volume depletion (VD) group. On the other hand, the cisplatin (CDDP) group shows loss of brush border membranes, obvious dilation of many tubules, and interstitial inflammatory infiltration. (b) Renal pathological score was significantly higher in the CDDP group than control and VD groups. Data are given as means±SD. (n = 4 for each group). *, p < 0.05, vs. the control group; †, p < 0.05, vs. the VD group; magnification, × 400.

H&E-stained kidney sections showed normal histology in the control group and near normal or mild brush border loss in the VD group (Fig 3(a)). On the other hand, the CDDP group showed loss of brush border membranes, obvious dilation of many tubules, nuclear loss and interstitial inflammatory infiltration. Index of histological damage was higher in the CDDP group than the control and VD group (Control, 11.3±2.6; VD, 13.8±3.4; CDDP, 33.8±4.5; P = 0.0016; Fig 3(b)).

#### Semiquantitative immunoblotting and renal expression of Klotho, S100A8/A9, and NGAL in pre-renal and intrinsic AKI models

**Fig 4:**
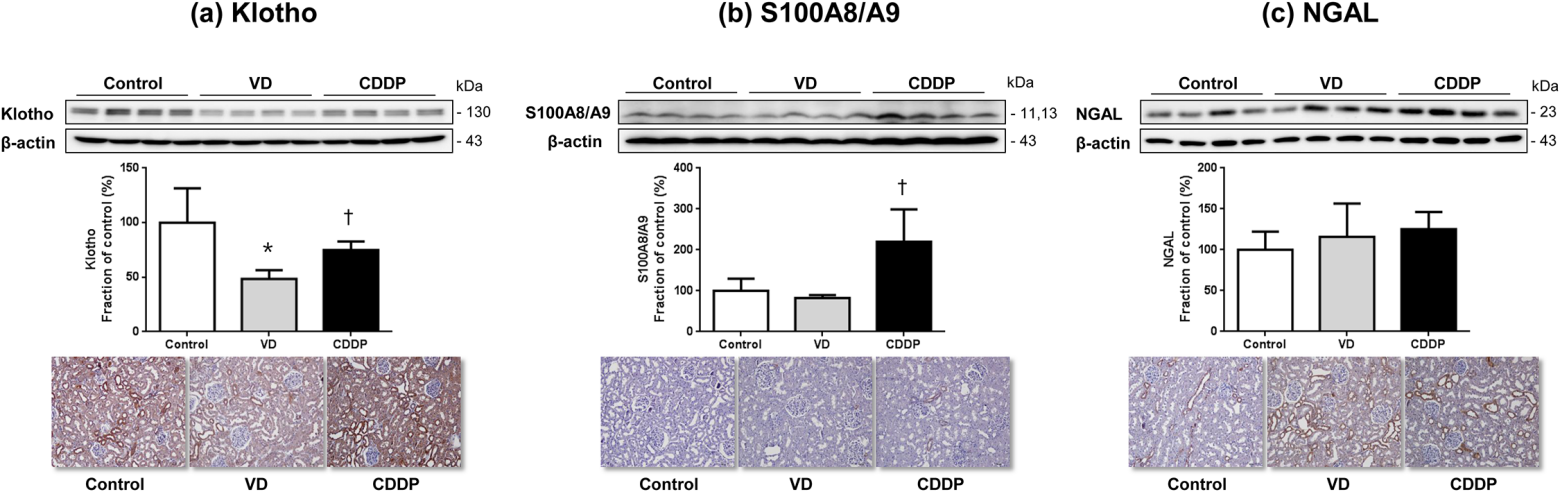
Semiquantitative immunoblotting and immunohistochemistry of the kidney from the acute kidney injury model. (a) Renal Klotho abundance was reduced in the volume depletion (VD) group (48%) versus the control (100%) and cisplatin (CDDP) groups (74%). Immunohistochemical analysis also showed decreased renal Klotho labeling in kidneys from VD group. (b) S100A8/A9 abundance was increased in the CDDP group (220%) versus the control (100%) and VD groups (83%). Immunohistochemical analysis also showed increased renal S100A8/A9 labeling in kidneys from CDDP group. (c) Neutrophil gelatinase-associated lipocalin (NGAL) abundance and labeling showed no significant difference between the VD and CDDP groups. Data are given as means±SD. *, p < 0.05, vs. the control group; †, p < 0.05, vs. the VD group; magnification, × 200.

As shown in Fig 4(a), the semiquantitative immunoblotting of the kidney homogenates showed a significant decrease in renal klotho protein in VD group (100% of those in control group; 48% of those in VD group; 74% of those in CDDP group, respectively) compared with the control and CDDP groups. Immunohistochemical analysis also showed decreased renal Klotho labeling in kidneys from VD group. As can be seen in Fig 4(b), S100A8/A9 in the CDDP group significantly increased (100% of those in control group; 83% of those in VD group; 220% of those in CDDP group, respectively) compared with that in the control and VD groups. This result was confirmed by immunohistochemistry showing increased labeling of renal S100A8/A9 in kidneys from CDDP group. As shown in Fig 4(c), NGAL protein levels and renal NGAL expression did not appear to differ between the VD and CDDP groups. These results were consistent with the ELISA results.

#### S100A8/A9 expression in inflammatory cells in intrinsic AKI models

**Fig 5:**
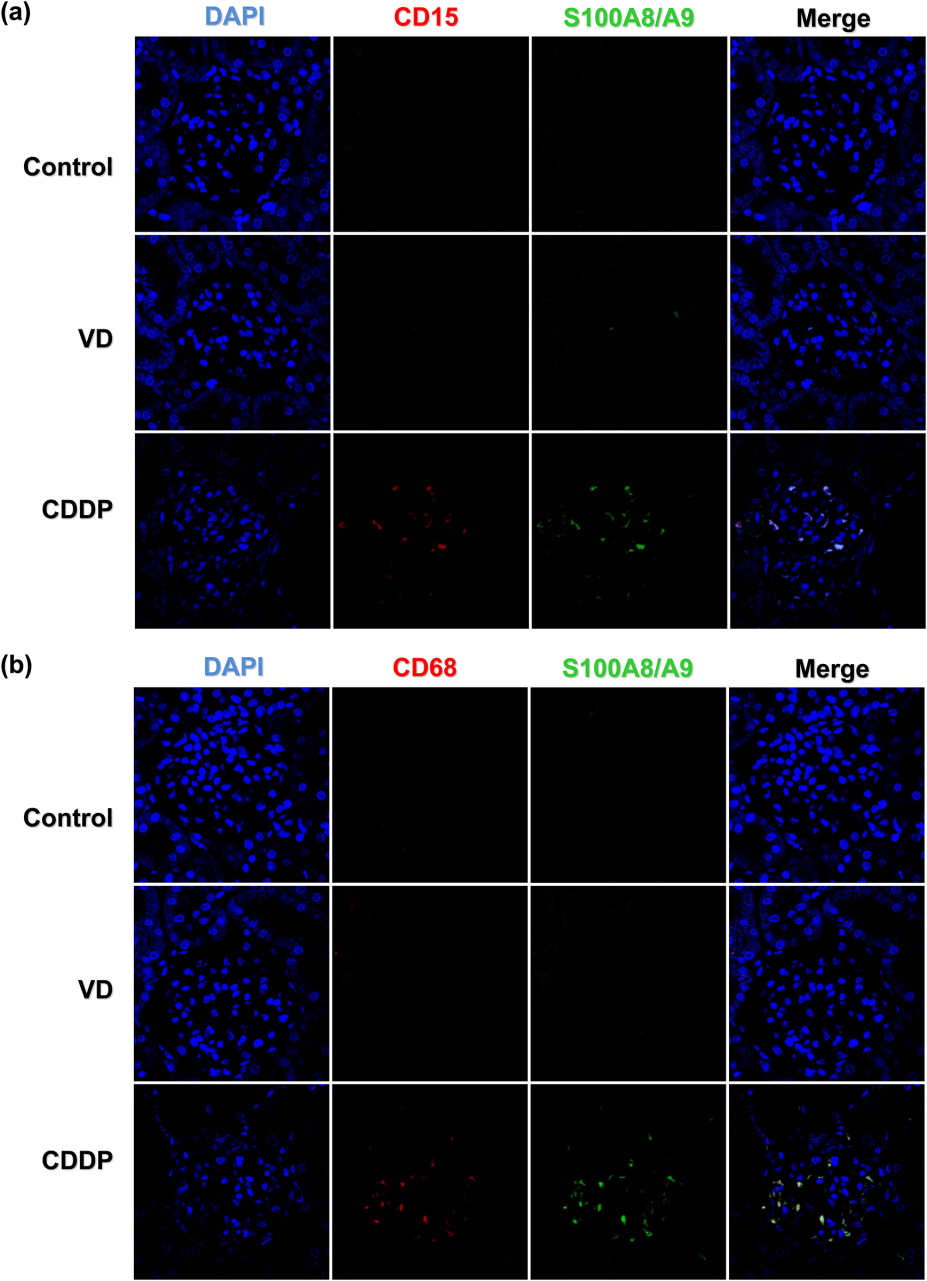
Immunofluorescence study to demonstrate the presence of S100A8/A9-positive cells in the kidney from an acute kidney injury model. Immunofluorescence study for neutrophils (CD15), monocytes/macrophages (CD68), and S100A8/A9-staining cells in an acute kidney injury model demonstrating that S100A8/A9, CD15, and CD69 staining were found frequently in the CDDP group which might reflect the inflammatory damage in this group. S100A8/A9 staining appeared to correlate with areas of CD15 (a) and CD68 staining (b). These findings also suggest that increased S100A8/A9 expression reflects the infiltration of inflammatory cells in intrinsic AKI. Magnification, × 400 in (a) and (b)

To evaluate the mechanism underlying the elevation of S100A8/A9 in the intrinsic AKI group, we examined inflammatory cells in an immunofluorescence study. As expected, intra-renal expression of S100A8/A9 was found more often in the CDDP group which might reflect the inflammatory damage in this group. In addition, intra renal infiltration of CD 15^+^ neutrophils and CD 68^+^ macrophages were found frequently in the CDDP group, and the area of distribution was consistent with that of S100A8/A9 (Fig 5(a) and 5(b)). These findings also suggest that increased S100A8/A9 expression reflects the infiltration of inflammatory cells in intrinsic AKI. These inflammatory cells were detectable in the glomerular and tubule-interstitial space.

### Clinical study

#### Patient characteristics

In total, 61 patients were enrolled in the study. Of them, 42 patients had pre-renal AKI and 19 had intrinsic AKI (Table 2). The severity of AKI, as measured by the RIFLE and AKIN criteria, did not significantly different between the pre-renal and intrinsic AKI group (P = 0.121 by RIFLE criteria, P = 0.172 by AKIN criteria). Serum creatinine was not different between the groups (3.3±2.4 mg/dL in pre-renal AKI, 2.6±1.3 mg/dL in intrinsic AKI; P = 0.296). However, BUN levels were higher in pre-renal AKI than intrinsic AKI patients (59.7±31.1 mg/dL in pre-renal AKI, 42.6±20.4 mg/dL in intrinsic AKI; P = 0.049). Age, gender, and other laboratory findings were not significantly different between the groups. Calcium channel blocker use was more common in intrinsic than pre-renal AKI patients (P = 0.042). Other medications were prescribed similarly in both groups.

**Table 2:** Baseline characteristics of acute kidney injury patients.

	Pre-renal AKI	Intrinsic AKI	*P*
	(*n* = 42)	(*n* = 19)	
Age	63±18	68±12	0.224
Male, *n* (%)	24 (57.1)	8 (42.1)	0.407
RIFLE Criteria			0.136
Risk, *n* (%)	13 (31)	3 (15.8)	
Injury, *n* (%)	11 (26.2)	10 (52.6)	
Failure, *n* (%)	18 (42.8)	6 (31.6)	
AKIN Criteria			0.221
Stage 1, *n* (%)	13 (31)	3 (15.8)	
Stage 2, *n* (%)	12 (28.6)	10 (52.6)	
Stage 3, *n* (%)	17 (40.5)	6 (31.6)	
Comorbidities			
Hypertension, *n* (%)	10 (23.8)	5 (26.3)	>0.999
Diabetes, *n* (%)	5 (11.9)	6 (31.6)	0.081
CVD, *n* (%)	11 (26.2)	4 (21.1)	0.757
Laboratory			
BUN, mg/dL	59.7±31.1	42.6±20.4	**0.049**
Creatinine, mg/dL	3.3±2.4	2.6±1.3	0.296
Peak creatinine, mg/dL	3.9±2.0	3.1±1.6	0.158
Hemoglobin, g/dL	10.8±2.3	9.9±1.6	0.220
WBC, ×10^3^/mm	12.964±6.719	10.611±4.983	0.153
Protein, g/dL	6.0±1.1	6.2±0.8	0.469
Albumin, g/dL	3.2±0.7	3.1±0.5	0.855
CRP, mg/dL	7.91±7.69	8.31±6.63	0.705
Medications			
ACEi/ARB, *n* (%)	3 (7.1)	4 (21.1)	0.080
Statin, *n* (%)	2 (4.8)	3 (15.8)	0.121
β-blocker, v (%)	4 (9.5)	5 (26.3)	0.091
CCB, *n* (%)	2 (4.8)	4 (21.1)	**0.042**
Diuretics, *n* (%)	9 (21.4)	5 (26.3)	0.486
Aspirin, *n* (%)	5 (11.9)	4 (21.1)	0.237

Abbreviations: AKI, acute kidney injury; RIFLE, risk, injury, failure, loss, and end-stage kidney disease; AKIN, acute kidney injury network; CVD, cardiovascular disease; BUN, blood urea nitrogen; WBC, white blood cell; CRP, C-reactive protein; ACEi, angiotensin-converting enzyme inhibitor; ARB, angiotensin II receptor blocker; CCB, calcium channel blocker.

#### Serum and urinary levels of Klotho, S100A8/A9, and NGAL levels for differentiating between pre-renal and intrinsic AKI patients

**Fig 6:**
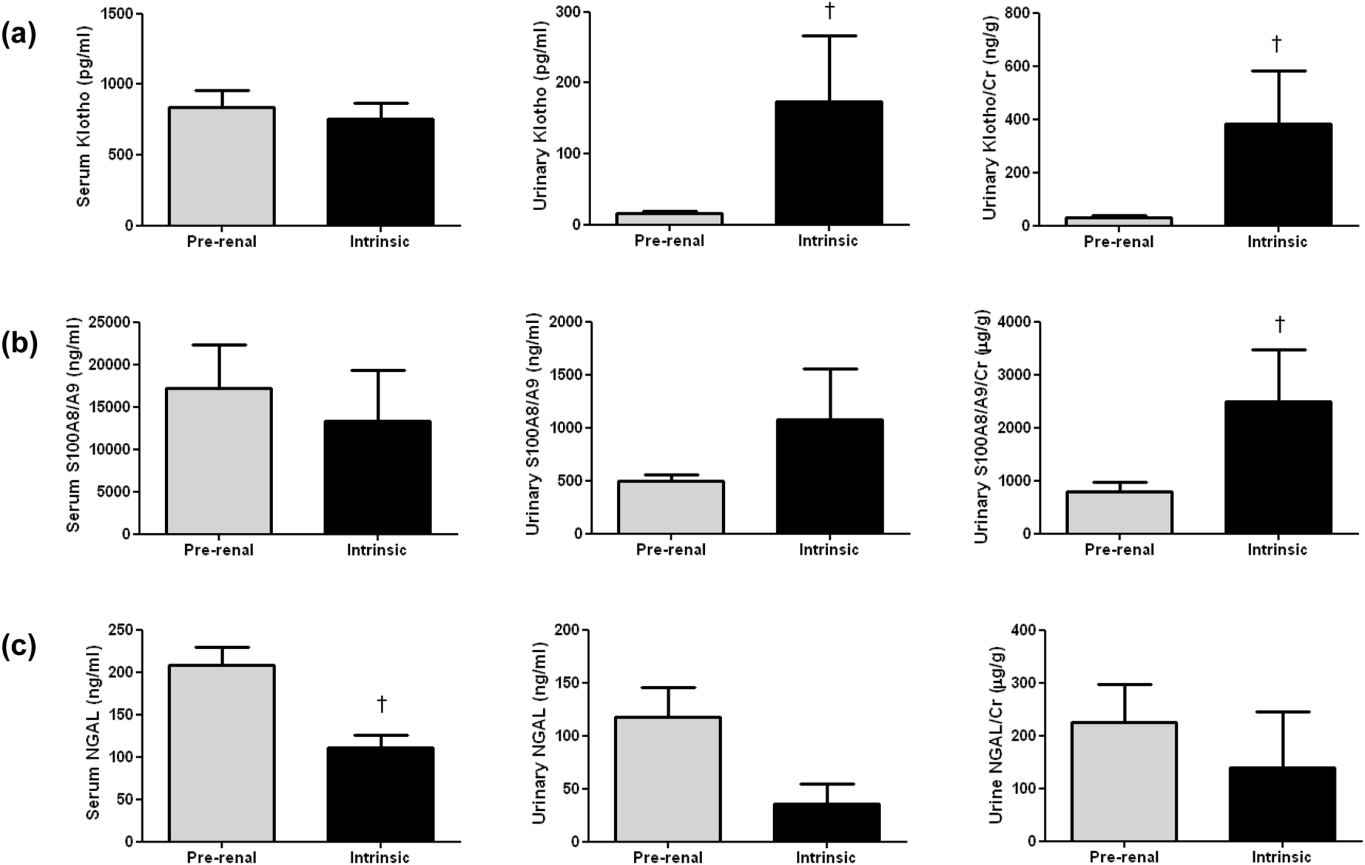
Serum and urinary Klotho, S100A8/A9, and neutrophil gelatinase-associated lipocalin (NGAL) concentrations in acute kidney injury patients. (a) Urinary Klotho and urinary Klotho/creatinine (Cr) decreased significantly in pre-renal AKI patients. (b) Urinary S100A8/A9/Cr was increased significantly in intrinsic AKI patients. (c) Serum NGAL was increased in pre-renal AKI patients. Data are given as means±SD. †, p < 0.05, vs. pre-renal AKI (*n* = 42 for pre-renal AKI patients, *n* = 19 for intrinsic AKI patients)

Serum Klotho levels were not significantly different in pre-renal (837.43±725.37 pg/mL) and intrinsic AKI (754.18±489.85 pg/mL, P = 0.986). Urine Klotho and the urine Klotho/creatinine ratio were decreased significantly in pre-renal versus intrinsic AKI patients (urine Klotho, 15.44±17.11 vs. 30.26±47.26 ng/g, P = 0.015; urine Klotho/creatinine ratio, 173.54±292.26 vs. 381.35±630.40 ng/g, P = 0.001; Fig 6(a)). Serum S100A8/A9 and urine S100A9/A9 concentrations were not significantly different in the two groups (P = 0.145 and 0.741, respectively). The urine S100A8/A9/creatinine ratio differed significantly, being higher in the intrinsic than the pre-renal AKI group (2494.88±3105.14 vs. 788.37±929.86 μg/g, P = 0.014; Fig 6(b)). Serum NGAL was higher in pre-renal than intrinsic AKI (208.30±132.51 vs. 111.05±63.33 ng/mL, P = 0.007). The urine NGAL and the NGAL/creatinine ratio, however, was not significantly different between the pre-renal and intrinsic AKI groups (P = 0.242 and 0.602, respectively; Fig 6(c)).

## Discussion

We showed that decreased urinary Klotho and increased urinary S100A8/A9 levels may be helpful in differentiating between pre-renal and intrinsic AKI. The urinary Klotho/creatinine ratio was decreased in a VD-induced AKI animal model and pre-renal AKI patients. The urinary S100A8/A9/creatinine ratio was elevated in a CDDP-induced AKI animal model and intrinsic AKI patients. Renal Klotho expression was decreased in a VD animal model, and renal S100A8/A9 expression was increased in a CDDP-induced intrinsic AKI model. Renal S100A8/A9 expression in the intrinsic AKI model may be related to the infiltration of neutrophils and macrophages.

A defect in the *klotho* gene results in a syndrome similar to aging [18], whereas overexpression of the *klotho* gene extends life span [19]. Klotho is expressed in the kidney, brain [18], pituitary, parathyroid [20], pancreas, ovary, testis, and placenta [18]. After its discovery in 1997 [18], subsequent studies have demonstrated various functions of the Klotho protein including anti-oxidation [21], anti-apoptosis, anti-senescence [22], promotion of angiogenesis [23], and inhibition of fibrogenesis [24]. In addition, full-length Klotho, a single-pass transmembrane protein, functions as a co-receptor for fibroblast growth factor-23, thus inhibiting phosphate absorption and promoting phosphaturia by inhibiting 1,25(OH)_2_ vitamin D3 synthesis, which may contribute to the prevention of vascular calcification [25,26]. Soluble Klotho modulates Na-phosphate co-transporter-2a [25], renal epithelial calcium channel [27], and the renal outer medullary potassium channel 1 [28]. Animal studies have shown transient renal Klotho deficiency in AKI under a variety of conditions, including hypovolemia [8], nephrotoxins such as CDDP and folic acid [29], ischemia-reperfusion injury [30], lipopolysaccharides [8], and ureteric obstruction [24]. However, there is limited data on whether Klotho may differentiate functional loss from structural damage in the kidney.

Our study demonstrated that serum and urinary Klotho levels significantly decreased in the VD-induced AKI model versus the CDDP-induced AKI model. In the proof-of-concept human study, urinary Klotho concentrations and the urinary Klotho/creatinine ratio were also decreased in the intrinsic AKI group compared to the pre-renal AKI group. These findings suggest that decreased urinary Klotho and the Klotho/creatinine ratio may represent loss of renal function. The results of this study are in accordance with previous observations, showing downregulation of Klotho expression in dehydration [31]. However, the current study differed from a study that showed downregulation of Klotho in a CDDP-induced AKI animal model [29]. That study did not measure serum or urinary Klotho, but only reported renal Klotho protein expression and *KL* mRNA transcripts. Moreover, only a mouse model was examined. Further studies are needed to determine whether the temporal changes in urinary Klotho during the time course of AKI may alter these findings, and whether the degree of urinary Klotho could reflect the degree of functional loss.

S100A8/A9 is calcium- and zinc-binding protein, consisting of a heterotetramer of S100A8 and S100A9 [32]. It is predominantly derived from neutrophils [33], but can also be produced by monocytes, activated macrophages, dendritic cells, and mucosal squamous epithelium [34]. S100A8/A9 is an activator (mediator protein) of the innate immune system and an amplifier of inflammatory activity via activation of Toll-like receptor 4 [35]. S100A8/A9 is an excellent biomarker of inflammatory processes, such as rheumatoid arthritis and juvenile idiopathic arthritis [36]. In addition, elevated S100A8/A9 plasma concentrations are an independent risk predictor for cardiovascular events in both healthy individuals [37] and patients with acute coronary syndrome [38]. It is also useful as a candidate for the detection of unstable versus stable coronary artery plaques [39]. Increased expression of S100A8/A9 has also been recently identified in various human cancers [40,41]. In the field of gastroenterology, fecal S100A8/A9 is a well-known biomarker for differentiating between inflammatory bowel disease and irritable bowel syndrome (a functional disorder without inflammation). Because there is no epithelial inflammation in irritable bowel syndrome, fecal S100A8/A9 concentrations are low. However, they are highly elevated in inflammatory bowel disease. Based on the absence of inflammatory damage in pre-renal AKI, it may be that the S100A8/A9 level does not increase in pre-renal AKI, whereas it is elevated in intrinsic AKI.

In the present study, animal experiments indicated that serum and urinary S100A8/A9 concentrations were elevated in CDDP-induced AKI versus the control groups, whereas there was no significant change in the VD group. In the proof-of-concept human study, the urinary S100A9/A9/creatinine ratio was also elevated in the intrinsic AKI group versus the pre-renal AKI group. Immunohistochemistry and confocal microscopy findings suggested that S100A8/A9 expression was increased in intrinsic AKI, and its expression was from the recruited inflammatory cells, including neutrophils and macrophages at the glomerular and tubule-interstitial space. These findings suggested that the increased urinary S100A8/A9/creatinine ratio represented structural damage and enhanced inflammatory activity of the kidney. Larger multicenter studies are needed for further validation of the use of S100A8/A9 in heterogeneous patient populations and for defining cut-off values for differential diagnosis and outcomes of AKI. Our results were consistent with those of other studies suggesting that urinary S100A8/A9 concentrations can be used to differentiate between intrinsic and pre-renal AKI [12,42].

Human NGAL was originally recognized as being bound to gelatinase in specific granules of the neutrophil. NGAL is synthesized during granulocyte maturation in the bone marrow [43], and is normally expressed in several human tissues, including the kidneys, lungs, prostate, uterus, stomach, and colon [44]. NGAL expression is induced markedly in injured epithelia in the setting of inflammation or malignancy [45]. NGAL is one of seven genes that show increased expression within the first several hours following ischemic or CDDP-induced nephrotoxic renal injury in animal models [46]. NGAL protein is highly expressed in kidney proximal tubule cells after ischemic or CDDP-induced renal injury and readily detected in the blood and urine shortly after AKI.

NGAL is one of the most widely studied and most promising biomarkers of AKI to date. It has been investigated in various clinical settings of AKI, such as after cardiopulmonary bypass and cardiac surgery [47], sepsis-associated AKI [48], contrast-induced AKI [49], in critically ill patients [50], in patients admitted to the emergency department [51], and AKI following kidney transplantation [52]. Moreover, previous studies showed that urinary NGAL discriminates intrinsic AKI from normal renal function, pre-renal AKI, and CKD in the emergency department [51] and effectively distinguishes pre-renal from intrinsic AKI in all hospitalized patients [11].

In the present study, serum NGAL and urinary NGAL/creatinine were higher in the intrinsic AKI animal model; however, serum NGAL was higher in pre-renal AKI patients. We suggest that this may be affected by the somewhat higher serum creatinine concentration among pre-renal AKI patients than intrinsic AKI, although it was not statistically significant. In addition, the proportion of stage 3 AKI patients by the AKIN criteria also tended to be higher in prerenal AKI than intrinsic AKI. Because previous reports have shown that urinary NGAL is correlated with the degree of renal damage [47], this different degree of renal dysfunction may affect the level of NGAL in both types of AKI patients. Further larger-scale studies are needed to determine the discriminative role of NGAL in AKI patients.

The attenuation of urinary Klotho and increase in urinary S100A8/A9 may contribute to discriminating between pre-renal and intrinsic AKI. The increased S100A8/A9 may indicate an activated immune response and inflammation in intrinsic AKI. Further studies are needed to assess whether biomarker combinations are likely to improve our ability to predict the cause of AKI and its outcomes and to validate the sensitivity and specificity of these biomarker combinations in larger, heterogeneous patient populations.
